# Migraine in neurological department of fann teaching hospital in Dakar

**DOI:** 10.1186/1129-2377-14-S1-P107

**Published:** 2013-02-21

**Authors:** LB Seck, M Ba, K Toure, A Thiam, AG Diop, NM Ndiaye

**Affiliations:** 1 Neurological Department - Fann Teaching Hospital, Senegal; 2 Neurological Department - Fann Teaching Hospital, Senegal

## Introduction

Migraine is the most frequent primary headache, one of the main complaint of our neurological out-patients department, but it has not been studied enough in our structure.

## Purpose

We aimed to assess clinical features of migraine in a sub-saharian teaching hospital.

## Methods

We carried out a prospective study in the out-patient department of the neurological service of Fann teaching hospital, in Dakar.

## Results

One hundred patients were collected, aged from 9 to 64 years with sex-ratio 5.25. It was migraine without aura in 83% of patients and with aura in 17%. Triggering factors were mainly psychical, climatic, hormonal, food, fatigue, physical effort, sensorial. The pain was hemicranial in 73%, throbbing in 82%, tightening or itching in 18%. Headache duration varied from 4 to 72 hours in 75%, less than 4 hours in 10%, more than 72 hours in 15%. Pain intensity was mild for 2%, moderate for 49% and severe for 49%. It occured once a day to less than once a month. Photophobia was found in 78%, nausea in 48%, vomiting in 33%. Aura was visual, psychical or sensorial. All patients benefited from treatment of acute pain, while 83% underwent permanent treatment in addition. Fifty eight per cent of women who had already been pregnant reported improvment during pregnancy.Of yhe whole sample, 59% had never seen a doctor for their migraine, using self-treatment or not pain-killer at all. Fifty three per cent of patients experienced a good outcome, while it was sationary for 31%, 17% being lost sight.

## Conclusion

Even if it is less studied in Africa, and is supposed to be less frequent in black people [1], migraine keeps its classical features. It considerably alter quality of life [2,3] but yet tend to be neglected by patients who do not care enough about it [3].
